# Chinese Herbal Medicine in Treating Primary Sjögren's Syndrome: A Systematic Review of Randomized Trials

**DOI:** 10.1155/2012/640658

**Published:** 2012-08-27

**Authors:** Hui Luo, Xinxue Li, Jianping Liu, Flower Andrew, Lewith George

**Affiliations:** ^1^Center for Evidence-Based Chinese Medicine, Beijing University of Chinese Medicine, Chaoyang District, Beijing 100029, China; ^2^Center for Research on Complementary and Alternative Medicine, University of Southampton, Primary Care, Southampton SO16 5ST, UK

## Abstract

*Background*. There is no curative treatment for primary Sjögren's syndrome (PSS). Chinese herbal medicine (CHM) is widely used in the treatment of PSS in China. *Objective*. To evaluate the effectiveness and safety of CHM for PSS. *Methods*. PubMed, Cochrane Library, China Knowledge Resource Integrated Database, Chinese Biomedical Database, Wanfang Data, and the Database for Chinese Technical Periodicals were searched for randomized controlled trials (RCTs) of CHM or CHM plus conventional medicine for PSS compared with placebo or conventional medicine. RevMan 5.0.17 was employed to conduct data analyses and assess homogeneity. Statistical models were chosen according to heterogeneity. *Results*. A total of 52 RCTs were included. The overall methodological quality of included trials was low. 49 trials reported response rates, of which 32 found significant improvements favoring CHM treatment against controls; 20 trials reported lacrimal function by Schirmer test scores, of which 16 trials reported a significant difference favoring CHM treatment. 21 trials reported salivary function by salivary flow rate, of which 10 reported significant favorable effects of CHM treatment. Other trials found no difference. The reported adverse effects of CHM included nausea, diarrhea, and other minor digestive symptoms, but more frequent adverse effects occurred in conventional medicine groups. *Conclusions*. Preliminary evidence from RCTs suggests the effect of CHM is promising for relieving symptoms, improving lacrimal and salivary function in PSS. However, the poor methodological quality of the included trials means that further well-designed, multicentered, larger trials are needed.

## 1. Introduction

Sjögren's syndrome, also known as “Mikulicz's disease” and “Sicca syndrome,” is a diffuse connective tissue disease in which immune cells attack and destroy the exocrine glands that produce tears and saliva [1]. Clinical symptoms of the disorder typically involve dryness of the mouth and eyes [2]. In addition, Sjögren's syndrome may affect other exocrine glands and organs of the body and cause multisystem signs and symptoms such as fatigue and joint pain. If a patient manifests the above symptoms together with positive blood tests for inflammatory and autoimmune markers, and other connective tissue diseases are eliminated, it suggests a diagnosis of primary Sjögren's syndrome (PSS) [3]. Further diagnostic confirmation can be obtained by the Schirmer test that measures tear production and biopsy of the salivary glands. Primary Sjögren's syndrome has developed into a global disease. It is estimated to affect as many as 3 million people in the USA alone making it one of the most common autoimmune rheumatic diseases [4]. In Europe, the prevalence of Sjögren's syndrome in the UK is estimated at approximately 0.33% [5], Greece 0.09% [6], and Slovenia 0.6% [7]. In China, the prevalence is 0.3%. Sjögren's syndrome incidence rates peak at age 45 to 50, and nine out of ten patients are women. Sometimes, it occurs in children [1, 8].

There are currently no known curative treatments for Sjögren's syndrome. Management is symptomatic and the most common treatments are prednisone, immunosuppressant, and symptomatic support, with the aim of relieving clinical symptoms and preventing organs being damaged by disease progression. Moisture replacement therapies such as artificial tears can relieve the symptoms (dry eyes) and corticosteroids or immunosuppressive drugs may be prescribed to control severe complications [1, 9]. These treatments are based on the experience of clinical physicians, expert opinion, and very limited clinical research. In 2010, a systematic review of treatment for PSS published in JAMA indicated the very limited evidence available for commonly used drugs and there is almost no evidence for any treatment for patients who do not respond to first-line therapies. We need more research for this relatively common and disabling condition if we are to provide effective, evidence-based, interventions [10].

There is no record of Sjögren's syndrome as such in the classical literature of traditional Chinese medicine (TCM), but knowledge of its clinical manifestations and symptomatic treatment can be traced back over 2000 years. Modern TCM researchers have conducted clinical trials on Sjögren's syndrome. A systematic review of these trials from 1997 to 2010 analyzed and evaluated randomized controlled trials of the Chinese herbal medicine (CHM) of treatment of Sjögren's syndrome [11]. However, the systematic review was written in Chinese and published in a Chinese journal and trials that recruited both primary and secondary Sjögren's syndrome patients or patients with severe complications were included in the paper. The methodological quality of the included trials at this point in time was generally poor, but over the past year 10 new RCTs have been published with a substantially improved methodological quality. Therefore, the objective of this systematic review is to appraise existing RCTs on CHM for PSS and provide an up-to-date evidence-based evaluation on the effectiveness and safety of CHM for PSS.

## 2. Methods

### 2.1. Eligibility Criteria

RCTs of CHM treating PSS were included, with no limitations on language or publication format. Trials were eligible when the study participants were patients with PSS, and there were no limitations on the participant's age, gender, and nationality. Interventions included any form of CHM (including prescribed formulae, patent medicines, herbal decoctions, herbal extracts and herbal injections) based on syndrome differentiation, and treatment, used as a sole treatment or in conjunction with conventional therapies. Control treatments included standard conventional treatment, placebo, and waiting list controls. Outcomes included total effectiveness rate (response rate), lacrimal function, salivary gland function, quality of life, and adverse events.

Trials were excluded if any of the following were identified: (1) The study population included patients with secondary Sjögren's syndrome, since this could not be differentiated from PSS; (2) if they involved the treatment of complications of PSS such as severe hepatic, renal damage or hematological damage; (3) if information about the participants or intervention was not clearly reported; (4) if controlled treatment included any use of CHMs as, in this case, it would be impossible to evaluate the specific effects of the intervention.

### 2.2. Information Sources

Both Chinese databases and English databases were searched, including China Knowledge Resource Integrated Database (CNKI), the Database for Chinese Technical Periodicals (VIP), Chinese Biomedical Database (CBM), Wanfang Data, PubMed, and the Cochrane Library (2012, Issue 2).

### 2.3. Searching

In the Chinese databases, we employed Sjögren's syndrome and random* as the main search terms without limitation on the modalities CHM employed. We searched PubMed by using the MeSH term Sjögren's syndrome with the following restrictions: humans as study participants, RCTs, or meta-analysis. When searching the Cochrane Library, we used Sjögren's syndrome as the key search word. We searched all articles on treatment for PSS published before February 10, 2012. The search strategy for the databases was provided in Table 3.

### 2.4. Selection

We employed the trial selection methods described in the Cochrane Handbook for Systematic Reviews of Interventions, version (1) import the search results from different databases into the reference management software NoteExpress2 (2.6 version); (2) exclude irrelevant articles by reading titles and abstracts; (3) obtain the full papers for all possibly relevant trials; (4) exclude articles with duplicate publication; (5) contact the authors when the data was not available; (6) recheck identified articles according to the above steps; (7) include the final trials for the review [68].

### 2.5. Data Collection Process

Data from the included trials were extracted by two authors independently. Any discrepancies were resolved by referral to the original article and, if necessary, a third author was consulted. The following data were extracted from included trials: methodological components, participant characteristics, interventions and controlled treatments, and outcome measures.

### 2.6. Risk of Bias in Individual Studies

The Cochrane Collaboration's tool for assessing the risk of bias was used to evaluate the methodological quality of included trials [68], which covers six domains of bias: selection bias, performance bias, detection bias, attrition bias, reporting bias, and other bias. Judgments were reached according to the text description or summary of relevant trial characteristics for each item in the tool.

### 2.7. Summary Measures and Synthesis of Results

Dichotomous data (response rate) was presented as risk ratio (RR) and continuous data outcomes (including lacrimal function, salivary gland function, and quality of life) as mean difference (MD), both with 95% confidence intervals (CI). RevMan 5.0.17 was employed to conduct data synthesis. Homogeneity of risk ratio or mean difference in trials with unequal sample sizes within one type of comparisons was analyzed using I^2^ and Z values, and statistical models were chosen based on significance of heterogeneity.

## 3. Results

### 3.1. Study Selection

**Figure 1:**
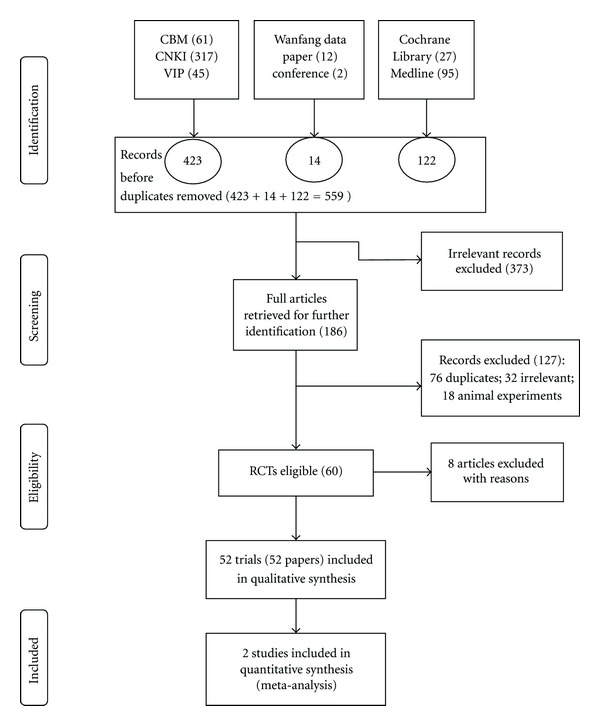
Flow-chart of study selection. PRISMA (preferred reporting items for systematic reviews and meta-analyses) flow-chart of study selection.

A total of 559 articles were found from the initial searches. After reading titles and abstracts, 60 full-text papers were retrieved and further 8 studies were excluded for the following reasons: 2 trials were excluded for involving patients with haematological complications [69] and kidney damage [70]; 3 trials were excluded due to CHM treatment in control group [71–73]; 1 trial was excluded due to being nonrandomized trial [74]; 2 trials were excluded due to duplication [75, 76]. Finally, 52 RCTs, which were published in Chinese, were judged to be eligible and included in the review [12–21, 23–61, 63–65] (Figure 1).

### 3.2. Study Characteristics

All the included trials were conducted in China, with a total number of 3,829 PSS patients, 12.9% of them were male (*n* = 495) and 85.5% were female (*n* = 3,272), and data on gender were not available in two trials (1.6%). The sample size of the included trials ranged from 20 to 256 participants with an average of 74 patients per trial. No trials reported how they estimated the sample size. The diagnostic criteria of PSS included the 2002 international classification of Sjögren's syndrome [66], the 1992 European diagnostic criteria [67], and other foreign or Chinese established criteria referred to in previous publications [1, 22, 62, 77, 78].

CHM therapy followed traditional treatment principles to nourish yin, moisten dryness and generate body fluids, replenish qi and blood, tonify the “lungs” and “spleen,” strengthen the “spleen” to remove dampness, activate blood to remove stasis, and clear fire poison. CHM treatment principles also included dispersing and reinforcing methods to treat diseases caused by excess and deficiency patterns. This reflects the features of a very mixed syndrome presentations in PSS patients. The herbal medicines included multicomponent decoctions, patent medicines (pills and tablets), concentrated herbal granules, and herbal injections. Twenty trials tested patent medicines and fixed formula granules, while 32 trials tested individualized herbal decoctions, permitting modified formulae according to individual participant's TCM syndrome. Conventional treatments included (1) symptomatic management, such as artificial tears, oculenta, artificial saliva, bromhexine, ambroxol hydrochloride, and parasympathomimetic alkaloid; (2) corticosteroids, such as prednisone; (3) immunosuppressive drugs, such as methotrexate, hydroxychloroquine, and cyclophosphamide; (4) other treatment, such as thymosin, vitamins, antibiotics, transfer factor, and nonsteroidal anti-inflammatory drugs.

All the included trials were classified into four comparisons according to the interventions: (1) CHM versus placebo (*n* = 2); (2) CHM versus conventional medicine (*n* = 19), in which 2 trials compared CHM plus placebo of conventional medicine to conventional medicine plus placebo of CHM [37, 65]; (3) CHM plus conventional medicine versus conventional medicine (*n* = 28), in which one trial compared CHM plus conventional medicine to conventional medicine plus placebo of CHM [25]; (4) CHM plus acupuncture versus conventional medicine (*n* = 3).

48 different CHMs were tested in 52 trials. Two patent medicines and one herbal injection, Jinju Qingrun capsule [12, 25], Xuefu Zhuyu oral liquid [27, 31, 35, 44, 46] and Glycyrrhizin Compound injections [44, 46], were studied in three and two trials, respectively. Due to the significant heterogeneity of the interventions, only two trials on Jinju Qingrun capsule could be pooled in a meta-analysis [12, 25].

Outcome measurements included response rate (total effectiveness rate), salivary gland function, lacrimal function, laboratory findings, TCM syndrome evaluation scores, and adverse events.

The response rate was a composite outcome index used throughout these trials, integrating factors from symptoms, signs, and laboratory findings [79]. The effect was considered positive when (1) clinical symptoms of dry mouth and eyes were markedly improved; (2) the value of Schirmer test and salivary flow rate were markedly increased. If there was no change evident in clinical symptoms or lab testing, then it was considered to be ineffective.

Salivary gland function was tested by collecting saliva and determining the amount produced in a five-minute period, or by sugar-melt test. Lacrimal function was evaluated by Schirmer test to measure the production of tears. Laboratory findings of included trials included erythrocyte sedimentation rate (ESR), C-reactive protein (CRP), rheumatoid factor (RF), antinuclear antibody (ANA), anti-Sjögren's syndrome A antibody (anti-SSA), anti-Sjögren's syndrome B antibody (anti-SSB), and IgA, IgG, and IgM. No trial employed quality of life to evaluate clinical outcome. Adverse events were reported in 36 trials.

Information on participants, interventions, comparators, and outcomes reported in each trial was presented in Table 1; and detailed data from the outcome report was shown in Table 2.

### 3.3. Risk of Bias within Studies

Methods to generate allocation sequence were reported in 14 trials [12, 13, 15, 18, 19, 23–25, 33, 39, 40, 47, 52, 56]. These included random number tables, computer statistical software, and block randomization. One trial reported allocation concealment by using sealed opaque envelopes [65]. Blinding was employed in five trials [24, 25, 33, 37, 65]. In Yu [24] and Lian [33]'s trial, placebo of CHM decoctions was used in the control group. In Zhang [25], Zhong [37], and Wang [65] trials, real CHM and placebo of conventional medicines were used in the treatment group, while real conventional medicine and placebo CHM were used in the control group. Placebos were indistinguishable from the real treatment with respect to color, smell, and packaging in these trials. Another seven trials reported participant drop out and loss to followup [12, 25, 32, 33, 38, 47, 49]. Base-line data of participants in all included trials was comparable with no other risk of bias detected.

### 3.4. Effect Estimation

#### 3.4.1. Response Rate

The response rate was defined as numbers of participants in both treatment and control group who had global or partial symptomatic improvement, defined by either physician assessment or by laboratory tests. 94.2% (49/52) of the trials reported response rate but none of the trials reported the estimated effect using RR and 95% CIs. According to the primary data reported in their papers, we calculated the RR and 95% CIs of response rates, and the results showed that 65.3% (32/49) of the trials found a significant difference between CHM treatment and control groups. The overall estimates of effect within the four comparisons were:

*CHM versus placebo *(*n* = 2): two trials compared a single herbal formula to placebo and only one [33] found significant improvement (RR: 4.25, 95% CI: 1.76 to 10.29; *P* < 0.001), the other [24] used a cross-over randomized design, and no difference was found (RR: 1.0, 95% CI: 0.84 to 1.18; *P* = 0.72).

*CHM versus conventional medicine *(*n* = 19): all trials in this category reported the response rate and 63.2% (12/19) found significant differences favoring the CHM treatment over the control groups [19, 21, 23, 39, 40, 50, 52, 55, 57, 58, 63, 65] (Table 2). Conventional therapies included artificial tears, prednisone, methotrexate, cyclophosphamide, vitamin, and other symptomatic management.

*CHM plus conventional medicine versus conventional medicine* (*n* = 28): 26 trials in this category reported the response rate, of which 69.2% (18/26) found significant differences favoring the CHM treatment over the control groups [12, 16, 18, 20, 25–27, 29–31, 35, 37, 42–44, 48, 49, 61]. Pooled data of two trials [12, 25] found that Jinju Qingrun capsule plus eye drops was more effective than hydroxychloroquine sulfate plus eye drops (RR: 1.28, 95% CI: 1.02 to 1.62; *P* = 0.04).

*CHM plus acupuncture/acupressure versus conventional medicine *(*n* = 3): two trials in this category reported the response rate. One trial compared CHM plus acupuncture and conventional medicine with conventional medicine, and found a significant better effect from the combination therapy (RR: 1.96, 95% CI: 1.01 to 3.81;  *P* < 0.05) [64]. Another trial [54] compared CHM plus ear acupressure with conventional medicine but failed to find any significant difference (RR: 1.15, 95% CI: 0.93 to 1.43; *P* = 0.2).

In the above 49 trials, only one trial was assessed as having a low risk of bias [65], reporting adequate method of allocation concealment, double-blinding methods, and no withdrawals during the study. This trial compared a CHM decoction (*Radix Rehmanniae Recens*, *Liriope spicata*, *Leguminosae, Pseudostellaria heterophylla*, *Cornu Bubali*, *Radix Salviae Miltiorrhizae*) with hydroxychloroquine in treating PSS. The results showed that this herbal decoction demonstrated a superior effect to hydroxychloroquine in the improvement of eye and mouth symptoms (RR: 1.66, 95% CI: 1.08 to 2.56; *P* < 0.05).

#### 3.4.2. Lacrimal Function

A total of 20 trials employed the Schirmer test to determine whether the participants' eyes could produce normal tears to keep the eyes moist. Five out of seven found significant improvement favoring the CHM over the conventional medicine [32, 40, 45, 47, 58], and 83.3% (10/12) of trials found significant difference between the CHM plus conventional medicine, and the conventional medicine alone [13, 17, 20, 25, 30, 38, 46, 59–61]. In addition, Yang [53] found that a herbal formula plus acupuncture was superior to methotrexate for improving lacrimal function (RR: 2.98, 95% CI: 2.01 to 3.95; *P* < 0.00001).

#### 3.4.3. Salivary Gland Function

There were 21 trials that either used a salivary flow rate test or sugar-melt test to determine the function of salivary glands, of which 52.4% (11/21) trials found that there was a significant difference between treatment and control groups, which favored the CHM 2 trials compared CHM to conventional medicine [32, 50]; 8 trials compared CHM plus conventional medicine to conventional medicine alone [12, 13, 25, 46, 49, 59–61]; and 1 trial used CHM plus acupuncture compared to conventional medicine [53]. The other trials failed to find a significant difference between treatment and control groups. One trial measured the salivary flow rate and the other tested sugar-melted time, and the data could not be converted and synthesized. 

#### 3.4.4. Quality of Life

No trials used validated quality of life (QoL) measures, such as WHOQOL or SF-36, to evaluate clinical effectiveness. Among the included trials, one applied self-developed criteria to evaluate QoL, but found no statistical difference between the treatment and control group [23].

### 3.5. Adverse Effects

Adverse effects were reported in 19 trials [12, 13, 15, 17, 18, 20, 25, 32, 34, 37–39, 44, 46, 47, 51, 56, 57, 61], and another seven reported that no adverse effects occurred during the study [14, 23, 24, 31, 33, 49, 59]. The adverse effects reported in CHM treatment included nausea, abdominal pain, diarrhea, and other minor gastrointestinal symptoms. When conventional medicines were used, either as the sole intervention or in conjunction with CHM, adverse effects included vomiting, diarrhea, insomnia, rashes, blurred vision, edema, central obesity, increased ALT level, mild hepatic dysfunction, renal dysfunction, anemia, increased fasting blood glucose level, hypertension, hyperlipoidemia, and leucopenia occurred.

A funnel plot was not conducted due to clinical heterogeneity of the included trials.

## 4. Discussion

### 4.1. Analysis of Effectiveness and Safety

The data within this paper involving 52 trials cannot be synthesized into a meta-analysis because of their heterogeneity. The included trials report moderate effectiveness (overall response rate and function improvement on lacrimal and salivary gland) for PSS treatment for CHM when compared with conventional medicine or placebo. We evaluated the safety reports from the CHM: the adverse effects occurred in the CHM group appear less than those in the conventional medicine group.

There is no known cure in conventional medicine for PSS and this paper provides preliminary evidence that CHM may be a promising and safe intervention for this chronic long-term condition. However, there are important limitations in this paper that weaken the recommendation of CHM for their clinical use.

### 4.2. Limitations of the Systematic Review

#### 4.2.1. Methodological Quality of Included Trials

Almost all the randomized trials of CHM identified in this review evaluating treatment for PSS have a high risk of bias. Only 28.8% (15/52) of the included trials reported the randomization process and few trials reported allocation concealment or blinding, and no trial reported intention-to-treat analysis.

However, it is important to note that the methodological quality of RCTs in this field has shown signs of improvement over the last 3 years. Reports on the generation of allocation sequence account for 13.0% (3/23) of included trials published between 1997 and 2007, compared to 25.0% (4/16) in RCTs published between 2008 and 2009, and 61.5% (8/13) in RCTs published between 2010 and 2011. Application of blinding in study design accounted for 0% (0/23) of included trials published between 1997 and 2007, compared to 17.2% (5/29) that were published between 2008 and 2011. Reports of dropout rates and those lost to followup accounted for 8.7% (2/23) of included trials published between 2008 and 2011, compared to 17.2% (5/29) of trials published between 2008 and 2011. Nevertheless, the current quality of RCTs' in CHM is still unsatisfactory, because only one in 52 trials reported all the items in the Cochrane *Risk of Bias* tool adequately, while all other trials were of moderate or high risk of bias. Since most of the trials report positive results, and no difference could be found between trials published prior to 2008 and those with better quality studies published from 2008 to 2011 (see Table 2), we consider that this may suggest therapeutic benefit of CHM.

#### 4.2.2. Inconsistency and Heterogeneity of Response Rates

The majority (94.2%, 49/52) of included trials used response rate (total effectiveness rate) as the primary outcome measure. This is a composite outcome index, integrating factors from symptoms, signs, and laboratory findings. This subjective and vague outcome index is prone to bias and misinterpretation, particularly if there is insecure or no blinding. In the included trials, the procedure for determining effectiveness rate has not been standardized and different approaches to defining the response rate have led to an inconsistency and heterogeneity in the assessment of the efficacy of these interventions. Under these circumstances, although the results of most trials suggest CHM can improve “response rate” more significantly than standard conventional medicine or placebo, we must still remain highly circumspect about the specific effect of CHM on PSS.

#### 4.2.3. Complexity of Interventions

In accordance with TCM theory interventions were designed to be adapted according to the specific presentations of PSS, so they varied from trial to trial. In addition CHM was frequently used in conjunction with other treatments including conventional herbal medicine, placebo, and acupuncture. This complexity of intervention makes it difficult to use double blinding (patients and physicians). It also complicates an assessment of the effects of different components of the interventions. In these instances we can only report the effectiveness of a whole therapeutic system such as TCM rather than a single isolated treatment.

### 4.3. Other Limitations of Included Trials

*Diagnostic criteria*: diagnosis of PSS was not consistent across included trials. About 63% of the trials employed international criteria for diagnosis of Sjögren's syndrome, and the remaining trials cited domestic criteria or unestablished criteria. This may increase variations among participants.

#### 4.3.1. Treatment Duration and Follow-Up

 PSS is a systemic autoimmune disease and requires long-term care and treatment. Therefore, evaluation of the effect of an intervention should be based on long-term treatment and followup. However in the included 52 trials, only six trials continued follow up beyond the six-month treatment [19, 30, 38, 52, 55, 61], and a further four trials had a relatively short 3–6- months followup [38, 44, 49, 61]. This is insufficient for the assessment of the long term effectiveness of CHM.

#### 4.3.2. Quality of Life

 fatigue is a common symptom in patients with Sjögren's syndrome, and greatly influences patients' quality of life [80, 81]. The SF-36 has been widely used in this context [2, 81] but none of the Chinese studies employed a validated instrument for quality of life evaluation for PSS.

### 4.4. Recommendations for Further Research

Studies are beginning to, and must continue to, report randomization sequence generation and allocation concealment in detail and employ blinding in outcome measurement and evaluation as well as an intention to treat analysis and a clear description of dropout. If we want to study the specific efficacy of CHM interventions, double-blind and placebo controlled trials are necessary. However, due to the limited evidence available for conventional drugs most frequently used in PSS [10], it is reasonable to justify employing pragmatic clinical trials which either add CHM to conventional medicines or use CHM in a comparative effectiveness research versus conventional medicine rather than comparing CHM with placebo.

The Medical Research Council (MRC) guidelines for complex interventions highlighted that complex interventions might work best if tailored to local circumstances rather than being completely standardized [82]. Since the interventions were designed to adapt TCM syndrome differentiations to the specific characteristics of individual PSS presentations, and complex treatment “packages” were employed in these trials (including CHM, acupuncture as well as integrating CHM with conventional medicine), we suggest that a whole system approach should be adopted. This would involve using the various treatment modalities within TCM (a combination of CHM, acupuncture, and dietary and lifestyle advice) and comparing that with a package of conventional medicine treatment.

We recommend that the diagnostic criteria from the 2002 International Classification of Sjögren's syndrome be used for future TCM PSS research. We also recommend an extended course of treatment and followup time, using both quantitative and qualitative outcomes to make a proper assessment of the effectiveness of this intervention. In-validated outcomes such as “response rate” are unreliable, difficult to standardize and interpret, and should not be used.

## 5. Conclusions

52 RCTs were analyzed in this systematic review, testing various CHMs in the treatment of PSS. The findings from these trials suggest that CHM delivered either as a sole treatment or in conjunction with conventional medicine may be more effective than conventional medicine in managing PSS symptoms-with specific reference to lacrimal and salivary gland function. However, a high risk of bias in these studies and the heterogeneity of the CHM intervention and outcome assessment suggest that these positive findings must be interpreted with considerable caution; we cannot recommend any specific Chinese herbal medicines for clinical use. This preliminary evidence supports the continuing use and evaluation of individualized CHM as a potentially promising and safe intervention for this syndrome. We recommend that adequately powered and rigorously conducted further research should employ a variety of trial methodologies including double-blind placebo-controlled RCTs, pragmatic, and comparative equivalence trials, to investigate CHM treatment for PSS. If the evidence justifies it CHM can then be more widely recommended as a treatment for this common and troublesome condition.

## Figures and Tables

**Table 1:** Characteristics of included randomized controlled trials.

Study ID	Diagnostic criteria	Sample size	Mean age (years)	Gender (male/female)	Intervention	Control	Duration (months)	Followup	Outcome measures
Zhang 2011 [12]	2002 international*	59	38.0	3/54	Jinju Qingrun capsule	Hydroxychloroquine sulfate	3	No	Response rate, amount of tear secretion, salivary flow rate, improvement of symptoms and signs, ESR, IgG, IgA, IgM, *γ*-globulin, and adverse effect

Zheng 2010 [13]	2002 international*	60	38.0	Female	Runzao formula and control intervention	Prednisone, methotrexate, and symptomatic support	3	No	Response rate, amount of tear secretion, salivary flow rate, GB, CRP, RF, and adverse effect

Yin 2010 [14]	2002 international*	40	49.0	Female	Yangyin Qingre Jiedu decoction	Prednisone	1	No	Response rate, symptom score, Amount of tear secretion, WBC, RF, ESR, and adverse effect

Liu 2010 [15]	2002 international*	132	41.0	57/75	Yuquan pill, Shengmai injection, and control intervention	Muscarinic receptor agonist, prednisone, *tripterygium wilfordii* polyglycosidum, cyclophosphamide, and symptomatic support	1	No	Response rate and adverse effect

Li 2010 [16]	Self-made criteria	240	41.0	86/154	Qingre Quyu decoction and control intervention	Symptomatic support	6	No	Response rate

Huang 2010 [17]	2002 international*	61	59.1	7/54	Shenmai injection and control intervention	Anethol trithione tablets	0.5	No	Response rate, amount of tear secretion, salivary flow rate, and adverse effect

Hu 2010 [18]	2002 international*	64	46.2	Female	Ziyin Yangxue Qingre formula and control intervention	Hydroxychloroquine sulfate tablets	3	No	Response rate, symptom score, amount of tear secretion, salivary flow rate, sugar-melt test, CRP, ESR, IgG, IgA, IgM, and adverse effect

Xuan 2010 [19]	2002 international*	60	53.0	21/39	Shenglu Runzao decoction	Hydroxychloroquine sulfate tablets	12	No	Response rate, symptom score, RF, ESR, SS-A, and SS-B

He 2010 [20]	1992 Europe**	48	35.0	Female	Total Glucosides of Paeony and Hydroxychloroquine sulfate tablets	Hydroxychloroquine sulfate tablets	3	No	Response rate, ESR, IgA, IgM, *γ*-globulin, amount of tear secretion, salivary flow rate, and adverse effect

Xie 2010 [21]	Dong 1996 [22]	60	55.2/52.6	11/49	Yin-nourishing decoction	Prednisone, cyclophosphamide	1	No	Response rate, symptom score

Li 2010 [23]	2002 international*	40	54.1/48.6	1/39	Yushu-Dihuang decoction	Hydroxychloroquine sulfate tablets	2	No	Response rate, symptom score, self-made quality of life, and adverse effect

Yu 2010 [24]	2002 international*	61	52.1/55.4	5/56	Zi Zao Yin	Placebo	3	No	Response rate, symptom score, salivary flow rate, liver-kidney function, ESR, CRP, ANA, SSA, SSB, and IgG

Zhang 2009 [25]	2002 international*	100	40.6	6/89	Jinju Qingrun capsule, placebo of prednisone and symptomatic support	Prednisone, placebo of Jinju Qingrun capsule and symptomatic support	3	No	Response rate, symptom score, amount of tear secretion, salivary flow rate, ESR, IgG, IgA, IgM, *γ*-globulin, and adverse effect

Yang 2009 [26]	NA	168	53.7	33/135	Yangyin Shengjin Qingre Tongluo formula and prednisone	Prednisone and cyclophosphamide	3 to 6	No	Response rate

Wang 2009 [27]	2002 international*	50	45 to 78	5/45	Xuefu Zhuyu oral liquid and control intervention	Transfer factor oral liquid	3	No	Response rate

Wang 2009 [28]	1992 Europe**	60	58.0	6/54	Yangyin Jianpi Huoxue decoction, methotrexatum and hydroxychloroquine	Methotrexatum, hydroxychloroquine and brombexine	3	No	Scores of symptoms and signs, ESR, and CRP

Wan 2009 [29]	Dong 1996 [22]	60	28 to 73	7/53	Quzao decoction and control intervention	Brombexine and symptomatic support	2	No	Response rate

Su 2009 [30]	1992 Europe**	60	53.1	2/58	Yangyin Huoxue Shengjin formula and control intervention	Hydroprednisone and methotrexate	6	No	Response rate, symptom score, amount of tear secretion, sugar-melt test, ESR, IgG, IgA, IgM, and T lymphocyte subpopulation

Mao 2009 [31]	2002 international*	100	45 to 75	10/90	Xuefu Zhuyu oral liquid and control intervention	Transfer factor capsule and symptomatic support	3	No	Response rate and adverse effect

Lu 2009 [32]	2002 international* and TCM diagnosis [11]	58	42.6	4/54	Shengjin granules	Hydroxychloroquine	3	No	Response rate, symptom score, salivary flow rate, amount of tear secretion, ESR, CRP, TNF-*α*, ICAM-1, IgG, IgA, IgM, and adverse effect

Lian 2009 [33]	2002 international* and TCM diagnosis [11]	40	52.1	Female	Shengjin Runzao granules	Placebo	1.5	No	Response rate, improvement of symptoms and signs, ESR, IgG, RF, and adverse effect

Huang 2009 [34]	2002 international*	58	29 to 68	5/53	Yiqi Yangyin Huoxue formula and control intervention	Anethol trithlone tablets	1	No	Response rate and adverse effect

Gao 2009 [35]	2002 international*	126	30 to 78	11/115	Xuefu Zhuyu oral liquid and control intervention	Transfer factor capsule and symptomatic support	3	No	Response rate

Liu 2009 [36]	1992 Europe**	60	41.33/39.95	4/56	Maiwei Dihuang decoction	Artificial tear, bromohexine hydrochloride	3	No	Response rate, symptom score, ESR, ALT, AST, IgG, IgA, IgM, RF, anti-SSA, anti-SSB, SIL-2R, and amount of tear secretion

Zhong 2008 [37]	2002 international*	256	37.0	26/230	Chaihu Tongluo capsule, placebo of prednisone and methotrexate	Prednisone acetate, methotrexate, and placebo of Chaihu Tongluo capsule	3	No	Response rate, salivary flow rate, amount of tear secretion, ESR, CRP, A/G, Tb, IgG, IgA, IgM, and adverse effect

Feng 2008 [38]	2002 international*	78	47.4	Female	Total glycosides of paeony and control intervention	Methotrexate	9	Yes	Response rate, amount of tear secretion, sugar-melt test, ESR, *γ*-globulin, and adverse effect

Lv 2008 [39]	2002 international*	124	43.6	16/108	Jinyuan decoction	Brombexine and symptomatic support	3	No	Response rate, T lymphocytes (NK cells, CD_3_, CD_4_, CD_8_), IgG, IgA, IgM, and adverse effect

Han 2008 [40]	2002 international*	58	32.6	5/53	Xuanfei Bujin particle	Brombexine	3	No	Response rate, amount of tear secretion, corneal staining test, BUT test, *β*2-M, sICAM-1, sIL-2R, ESR, IgG, IgA, IgM, and improvement of symptoms

Wu 2007 [41]	Guidelines of China [1]	42	55.0	8/34	Maiwei Dihuang decoction and control intervention	Brombexine, thymopeptide and symptomatic support	2	No	Response rate and improvement of symptoms

Yan 2007 [42]	NA	56	Median: 55.2/53.6	6/50	Jiawei Shengmai drink and control intervention	symptomatic support	4	No	Response rate

Sun 2007 [43]	2002 international*	124	44.9	16/108	Qingli Shutong formula and control intervention	Brombexine and symptomatic support	3	No	Response rate and salivary flow rate

Shen 2007 [44]	NA	20	60 to 70	Female	Hydrocortisone injection and compound glycyrrhizin injection	Hydrocortisone injection and diammonium glycyrrhizinate injection	2 to 3	Yes	Response rate and adverse effect

Mao 2007 [45]	1992 Europe**	40	53.1	4/36	Yiqi Jianpi decoction	Prednisone	3	No	Response rate, symptom score, amount of tear secretion, salivary flow rate, sugar-melt test, ESR, CRP, RF

Li 2007 [46]	2002 international*	68	47.3	7/61	Compound glycyrrhizin injection and control intervention	Hydroxychloroquine, brombexine, and symptomatic support	1	No	Response rate, amount of tear secretion, tear break-up time, salary flow rate, *β*2-M, ESR, RF, IgA, IgG, IgM, and adverse effect

Zhou 2006 [47]	2002 international*	60	Median: 50/46.25	5/55	Qingzao Jiedu Yangyin Runzao formula	Prednisone and symptomatic support	3	No	Response rate, amount of tear secretion, salivary flow rate, sugar-melt test, IgG, and adverse effect

Zhou 2006 [48]	2002 international*	45	55.0	11/34	Dandi Qiongyu granules and control intervention	Brombexine, anethol trithione, thymopeptide, and symptomatic support	2	No	Response rate and symptom score

Shen 2006 [49]	Feng 1999 [50]	60	52.8	4/56	Runzao oral liquid and control intervention	Bromhexine and symptomatic support	3	Yes	Response rate, amount of tear secretion, salivary flow rate, sugar-melt test, rose bengal staining test, foci lymphocyte infiltrates, ESR, RF, *γ*-globulin, anti-SSA antibody, anti-SSB antibody, antinuclear antibody, and adverse effect

Niu 2006 [51]	1992 Europe**	40	33.4	1/39	Jianpi Huashi Qingre formula and control intervention	Prednisone and symptomatic support	1	No	Response rate, ESR, CRP, IgG, IgA, IgM, and improvement of symptoms and adverse effect

Chen 2006 [52]	1992 Europe**	60	52.8	5/55	Suangan Shengjin formula	Prednisone	6	No	Response rate, RF, and ESR

Yang 2005 [53]	Feng 1999 [50]	84	41.5	3/81	Yangyin Shengjin formula and acupuncture	Methotrexate	3	No	Response rate, amount of tear secretion, salivary flow rate, ESR, CRP, and IgG

Si 2005 [54]	TCM diagnosis of SATCM [55]	58	22 to 70	26/32	Runzao Tuiyi Mingmu decoction and auricular-plaster therapy	Prednisone and symptomatic support	1 to 3	No	Response rate

Liu 2005 [56]	2002 international*	60	Median: 48.5/48	5/55	Qingzao formula	Prednisone	3	No	Response rate, improvement of symptoms, amount of tear secretion, salivary flow rate, ESR, CRP, and adverse effect

Zhao 2003 [57]	Dong 1996 [22]	60	Median: 48/44	2/58	Qiju Dihuang decoction	Hydroxychloroquine and symptomatic support	NA	No	Response rate and adverse effect

Qian 2003 [58]	NA	72	NA	5/67	Jinxueyuan granules	Brombexine	3	No	Response rate, improvement of symptoms, sugar-melt test, amount of tear secretion, and ESR

Li 2003 [59]	Dong 1996 [22]	60	35 to 59	6/54	Shengmai injection and control intervention	Symptomatic support	0.5	No	Amount of tear secretion, salivary flow rate, and adverse effect

Wu 2002 [60]	Dong 1996 [22]	40	Median: 56	4/36	Ziyin Huoxue formula and control intervention	Thymosin and symptomatic support	2	No	Response rate, salivary flow rate and amount of tear secretion

Shen 2002 [61]	Feng et al. 1999 [50]	60	52.9	5/55	Liuwei Dihuang decoction, Zengye decoction, and symptomatic support	Bromhexine and symptomatic support	6	Yes	Response rate, salivary flow rate, amount of tear secretion, sugar-melt test, tear break-up time, rose bengal staining test, lymphocytes Infiltration of a labial gland, RF, ESR, anti-SSA antibody, anti-SSB antibody, antinuclear antibody, and adverse effect

Hu 2001 [55]	Manthorpe 1981 [62]	150	43.9	23/127	Zengye mixture formula	Brombexine and thymopeptide	6	No	Response rate, symptom improvement, amount of tear secretion, sugar-melt test, FL, ESR, ANA, RF, IgG, IgA, and IgM

Wang 2000 [63]	1992 Europe**	40	50.4	5/35	Runzao mixture formula	hydrochloride salt and symptomatic support	NA	No	Response rate

Feng 2000 [50]	Dong1996 [22]	44	34 to 65	1/43	Shengjin Runzao granules	Symptomatic support	1	No	Salivary flow rate

Zhou 1997 [64]	NA	50	52.0	12/38	Qiju Dihuang pill or Huanglian Shangqing pill or Maiwei Dihuang pill or Shihu Yeguang pill, fresh decoction of phragmites, Glycyrrhiza, and acupuncture	parasympathomimetic alkaloid and symptomatic support	NA	No	Response rate

Wang 2010 [65]	2002 international	57	Unclear	Unclear	Yiqi Yangyin quyu formula and placebo of hydroxychloroquine	Hydroxychloroquine and placebo of yiqi yangyin quyu formula	6	No	Sexual hormone, symptom improvement

Note. 2002 international*: 2002 international classification of Sjögren's syndrome proposed by the American-European Consensus Group [66]; 1992 Europe**: Preliminary criteria for the classification of Sjögren's syndrome by the European Community [67]; NA: not available.

**Table 2:** Trials evaluating Chinese herbal medicines.

Study ID	Sample size	Response rate RR (95% CIs)	Schirmer test MD (95% CIs) (mm/5 min)	Salivary flow rate test MD (95% CIs) (mL/min)	Adverse effects
CHM versus placebo					

Lian 2009 [33]	19/19	4.25 [1.76, 10.29]			NR
Yu 2010 [24]	30/31	1.03 [0.86, 1.24]		−0.57 [−1.75, 0.60]	NR

CHM versus conventional treatment						

Chen 2006 [52]	40/20	1.65 [1.04, 2.62]			NR
Feng 2000 [50]	34/10	6.76 [1.04, 44.06]		151.00 [46.32, 255.68]	NR
Han 2008 [40]	38/20	1.87 [1.13, 3.10]	2.98 [1.48, 4.48]		NR
Hu 2001[55]	100/50	1.31 [1.09, 1.59]		−10.40 [−14.21, −6.59]	NR
Huang 2009 [34]	32/28	1.23 [1.00, 1.51]			NR
Li 2010 [23]	130/110	1.24 [1.12, 1.37]			no AE
Liu 2009 [36]	30/30	1.67 [1.00, 2.76]			NR
Liu 2005 [56]	30/30	1.05 [0.78, 1.40]	0.26 [−1.12, 1.64]	−7011.40 [−7013.31, −7009.49]	C: 12 with central obesity, 2 with increased fasting blood glucose level, 1 with insomnia, 1 with hypertension, 1 with secondary fungus infection
Lu 2009 [32]	30/28	1.37 [0.98, 1.92]	2.12 [0.75, 3.49]	2.80 [1.94, 3.66]	T: 1 with diarrhea; C: 1 with blurred vision, 1 with pruritus
Lv 2008 [39]	74/50	1.35 [1.13, 1.61]			C: 2 with diarrhea
Mao 2007 [45]	20/20	1.38 [0.97, 1.97]	2.12 [0.75, 3.49]	−0.34 [−1.01, 0.33]	NR
Qian 2003 [58]	55/17	8.04 [2.18, 29.59]	2.12 [0.75, 3.49]	−6.05 [−8.42, −3.68]	NR
Wang 2000 [63]	30/10	2.42 [1.13, 5.18]			NR
Xie 2010 [21]	30/30	1.27 [1.01, 1.61]			NR
Xuan 2010 [19]	30/30	1.80 [1.23, 2.62]			NR
Yin 2010 [14]	20/20	0.94 [0.71, 1.25]	0.05 [−1.15, 1.25]		no AE
Zhao 2003 [57]	30/30	1.50 [1.03, 2.19]			gastrointestinal reactions: 10% versus 40% (*P* = 0.01); C: 3 with leucopenia, 2 with rashes
Zhou 2006 [47]	30/30	1.09 [0.84, 1.40]	2.33 [1.79, 2.87]	0.40 [−0.25, 1.05]	NR
Wang 2010 [65]	30/27	1.66 [1.08, 2.56]			NR

CHM plus conventional treatment versus conventional treatment					

Feng 2008 [38]	42/36	1.29 [0.91, 1.82]	1.25 [0.57, 1.93]	−7.12 [−10.61, −3.63]	T: diarrhea (11.9%), 1 withdrawal with severe diarrhea; C: 2 withdrawal with increased ALT level and hypoplasia respectively, rashes (5.6%)
Gao 2009 [35]	63/63	1.63 [1.24, 2.13]			NR
He 2010 [20]	26/22	1.79 [1.03, 3.11]	3.24 [1.94, 4.54]	−5.36 [−8.74, −1.98]	Blurred vision, abdominal discomfort, stomachache, bowel movement frequency: 11.5% versus 9.1%
Hu 2010 [18]	33/31	1.36 [1.02, 1.82]			T: 1 with abdominal pain and diarrhea; control group: 1 with nausea
Huang 2010 [17]	32/29	1.31 [1.00, 1.72]	1.46 [0.33, 2.59]		C: 3 with abdominal discomfort, diarrhea; 1 with increased ALT level
Li 2003 [59]	40/20		1.30 [0.54, 3.14]	135.70 [50.56, 220.84]	no AE
Li 2007 [46]	36/32	1.24 [0.97, 1.58]	2.26 [1.11, 3.41]	0.21 [0.18, 0.24]	T: 1 with edema; C: 1 with rashes, 2 with nausea
Li 2010 [16]	130/110	1.24 [1.12, 1.37]			NR
Liu 2010 [15]	67/65	1.09 [0.97, 1.24]			C: 2 with nausea, vomiting
Mao 2009 [31]	50/50	1.43 [1.11, 1.84]			NR
Niu 2006 [51]	20/20	1.31 [0.90, 1.89]			thrombocytopenia: 15% versus 35% (*P* = 0.15)
Shen 2002 [61]	30/30	1.69 [1.18, 2.41]	4.01 [3.06, 4.96]	0.25 [0.21, 0.29]	T: 1 with swelling parotid gland and increased ESR; C: 1 with distal renal tubular acidosis
Shen 2006 [49]	30/30	3.69 [2.76, 4.62]		0.23 [0.18, 0.28]	no AE
Shen 2007 [44]	10/10	1.91 [1.04, 3.50]			edema: 10% versus 40% (*P* = 0.15); hypertension: 10% with 30% (*P* = 0.28)
Su 2009 [30]	30/30	1.86 [1.24, 2.79]	0.85 [0.13, 1.57]	−5.73 [−9.45, −2.01]	NR
Sun 2007 [43]	74/50	1.35 [1.12, 1.63]		35.50 [−19.76, 90.76]	NR
Wan 2009 [29]	30/30	1.75 [1.24, 2.48]			NR
Wang 2009 [28]	30/30		−0.14 [−0.97, 0.69]		NR
Wang 2009 [27]	25/25	1.47 [1.03, 2.08]			NR
Wu 2007 [41]	22/20	1.44 [0.97, 2.14]			NR
Wu 2002 [60]	30/10	2.00 [0.92, 4.36]	2.40 [1.79, 3.01]	199.00 [107.42, 290.58]	NR
Yan 2007(50)	30/26	1.41 [1.01, 1.97]			NR
Yang 2009 [26]	85/83	1.36 [1.14, 1.62]			NR
Zhang 2009 [25]	49/46	1.19 [1.02, 1.39]	3.26 [2.28, 4.24]	435.46 [371.98, 498.94]	C: 2 with hepatic dysfunction, 1 with increased fasting blood glucose level, 1 with central obesity
Zhang 2011 [12]	29/28	1.51 [1.06, 2.15]	1.70 [0.82, 2.58]	0.80 [0.07, 1.53]	C: 1 with mild hepatic dysfunction
Zheng 2010 [13]	30/30	1.09 [0.84, 1.40]	3.71 [1.88, 5.54]	1.47 [1.18, 1.76]	T: 1 with nausea, 3 with stomachache; C: 2 with stomachache, 2 with diarrhea, 2 with increased ALT level, 1 with leucopenia; (*P* = 0.04)
Zhong 2008 [37]	128/128	1.17 [1.07, 1.29]			C: 1 with increased blood glucose level, 2 with hyperlipoidemia, 1 with hepatic and renal dysfunction
Zhou 2006 [48]	22/23	1.39 [1.01, 1.93]			T: 2 with abdominal swelling; C: 4 with obesity, 4 with stomachache, 2 with hypertension, 4 with insomnia

CHM plus acupuncture/acupressure versus conventional treatment					

Yang 2005 [53]	42/42		2.98 [2.01, 3.95]	2.39 [1.17, 3.61]	NR
Zhou 1997 [64]	34/16	1.96 [1.01, 3.81]			NR
Si 2005 [54]	38/25	1.15 [0.93, 1.43]			NR

Note. C: control group; T: treatment group; NR: not reported; AE: adverse effects.

**Table 3:** Search strategy.

Database	Search strategy
PubMed	“Sjögren's syndrome” (mesh) and (“humans” (MeSH terms) and (meta-analysis (ptyp) OR randomized controlled trial (ptyp))
Cochrane Library	“Primary sjögren's syndrome in record title in cochrane central register of controlled trials”
China Knowledge Resource Integrated Database (CNKI)	“Sjögren's syndrome in record title AND random* in all text”
VIP Database for Chinese Technical Periodicals (VIP)	“Sjögren's syndrome in record title AND random* in all text”
Wanfang Data	“Sjögren's syndrome in record title AND random* in all text”
Chinese Biomedical Database (CBM)	“Sjögren's syndrome in record title AND random* in all text”
